# Evaluation of the Effect of Distance Between Dental Abutments on the Accuracy of One‐Step and Two‐Step Impression Techniques With Polyvinyl Siloxane (PVS) Material

**DOI:** 10.1002/cre2.70100

**Published:** 2025-03-02

**Authors:** Seyyed Ahmad Ghoraishian, Mohsen Khataminia, Zahra Shahramian, Maryam Zare, Mina Mohaghegh

**Affiliations:** ^1^ Department of Prosthodontics Shiraz University of Medical Sciences Shiraz Dental School Shiraz Iran; ^2^ Shiraz University of Medical Sciences Shiraz Dental School Shiraz Iran; ^3^ Private Dentist Shiraz Iran

**Keywords:** interabutment distance, one‐step impression, PVS material, two‐step impression

## Abstract

**Objective:**

The aim of this study was to evaluate the effect of distance between dental abutments on the accuracy of one‐step and two‐step impression techniques with polyvinyl siloxane (PVS) material.

**Materials and Methods:**

Four master models were fabricated with different interabutment distances equal to one, two, three, and four premolar lengths 1–4 (L1, L2, L3, and L4). Seven one‐step impressions were taken from each master model using PVS impression material (Group A, *n* = 7). For two‐step impressions, a 1.5 mm polyethylene spacer was used over each master model, and impressions were taken (Group B, *n* = 7). Scans from the casts were superimposed over the master model scans. Accordingly, differences were measured and compared with statistical tests (Kolmogorov–Smirnoff test, Mann–Whitney test, and Friedman's test) to evaluate the effect of interabutment distance within each impression technique group and also compare the one‐step and two‐step impression techniques. *p* value < 0.05 was considered statistically significant.

**Results:**

A statistically significant difference was noted among the different edentulous areas in the one‐step impression technique group (Group A, *p* = 0.010). At edentulous span, a statistically significant difference was recorded (*p* = 0.047) with two‐step impression technique being more accurate compared to the one‐step impression technique. Friedman's pairwise analysis in Group A demonstrated a significance between one premolar and four premolar interabutment distance groups (*p* = 0.006).

**Conclusion:**

The accuracy of the one‐step impression is significantly affected by the increase in interabutment distance from one premolar to four premolar edentulous.

## 1 Introduction

The goal of dental science is to conserve health, function, and integrity of the dental arch of an individual as long as possible. Making an impression is an integral part of fixed prosthodontics. Accurate impression‐taking and the precision of impressions have always been a concern for dental clinicians (Aswani et al. 2020). One or more observable errors have been detected in many impressions. Several factors can affect impression accuracy, including impression technique and materials, gypsum/water ratio, vacuum hand mixing, type of impression tray, setting time, and accurate cast preparation (de Alencar Vasconcelos et al. 2020). A lack of accurate impressions can lead to biomechanical complications and marginal bone loss. If the impression is not precise, the prostheses may not fit properly, resulting in biomechanical issues such as uneven bite forces, excessive stress on neighboring teeth, and jaw joint problems. Additionally, inaccurate impressions can lead to marginal bone loss around dental implants, compromising the stability of the implant and resulting in further complications (Ender et al. 2019; Flügge et al. 2018).

Different materials and impression techniques were used to achieve high‐accuracy impressions. Despite advances in the materials and techniques for impression taking, there is still a need for further improvements in this area. An ideal impression material should meet certain requirements, such as easy manipulation, low thermal shrinkage, low polymerization shrinkage, dimensional accuracy, elastic recovery, nontoxicity, nonirritation, and reasonable cost. However, none of the available impression materials possesses all of these favorable properties simultaneously (Papadiochos et al. 2017; Moreira et al. 2015; Carvalho et al. 2018).

The interabutment distance is an important consideration in prosthodontics, as it impacts the design and stability of the dental prosthesis. A greater interabutment distance may require modifications to the design of the prosthesis to ensure proper fit and function. Additionally, the interabutment distance can affect the biomechanics of the prosthesis, influencing the distribution of forces and stresses on the supporting abutments (Shalileh et al. 2023).

During the 1970s, the introduction of polyvinyl siloxane (PVS) impression material revolutionized the market, establishing itself as the preferred choice due to its exceptional physical properties, ease of use, precise dimensional accuracy, and remarkable dimensional stability (Punj et al. 2017). Various conventional methods have been proposed to enhance the precision of PVS impressions. These include the utilization of dual‐phase one‐step addition silicone impression material, where both materials polymerize simultaneously, as well as the two‐step technique. In the two‐step technique, a putty material is initially employed as a custom tray, followed by the creation of a final impression using a silicone with lower viscosity (Khan and Mushtaq 2018; Azevedo et al. 2019).

Although the conventional impression‐taking method is widely used, it does have certain disadvantages, including the potential for infection transmission through the impression material (Afshari et al. 2020). Dental practitioners widely utilize advanced techniques like intra‐oral scanning to address the limitations of traditional impression‐taking methods (Dad et al. 2022). Nevertheless, the existence of toothless gaps amidst dental abutments may lead to increased disparities and less precise dental prostheses when intra‐oral scanning is employed in contrast to traditional techniques (Nouri et al. 2019). Therefore, the decision in the selection of the impression technique becomes very essential (Messias et al. 2019). Accordingly, the aim of this study was to evaluate the effect of distance between dental abutments on the accuracy of one‐step and two‐step conventional impression techniques with PVS material.

The null hypothesis was that the interabutment distance has no effect on accuracy of the one‐step and two‐step impression techniques. Additionally, the second hypothesis was that one‐step and two‐step technique has no effect on accuracy of impression results.

## 2 Materials and Methods

This in vitro study has received approval from the ethical committee of Shiraz University of Medical Sciences (IR.SUMS. DENTAL.REC.22471).

### 2.1 Model Teeth Preparation

**Figure 1:**
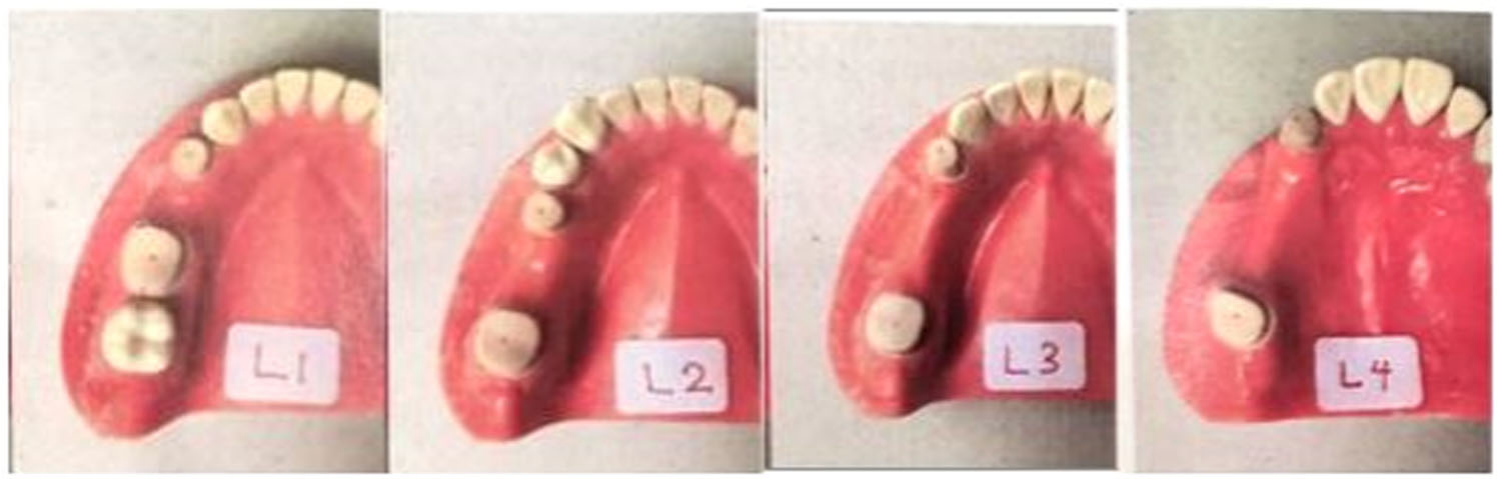
Four master models with corresponding one‐premolar (L1), two‐premolar (L2), three‐premolar (L3), and four‐premolar (L4) interabutment distances.

Four dentiforms were chosen, each with different numbers of missing premolar teeth. The TRIOS intra‐oral scanner from 3shape (Copenhagen, Denmark) was utilized to digitally scan the typodont teeth. Following the instructions provided by the manufacturer, a total of five digital impressions were captured. These intra‐oral scans were then transferred to the software and designed with a supragingival chamfer margin and a 7‐degree taper using the CAD/CAM machine known as dental designer‐3 shape‐trios, which was developed in Denmark in 2019. The designed models were subsequently constructed using stereolithography technology. The STL files were superimposed on the reference scans using the 3shape dental‐designer software. Markings were placed on the buccal, lingual, and proximal surfaces of the abutment teeth to serve as reference points for the superimposition process. The dentiforms were scanned using the 3Shape D810 Dental 3D Scanner (ID: 10738331) from Denmark and labeled as master models L1, L2, L3, and L4. From each master model, one‐step and two‐step impressions were taken using PVS Zhermack Elite HD+ impression material (Figure 1).

### 2.2 One‐Step Method

In the one‐step technique, the putty impression material (Silicone, Germany) was mixed manually for 30 s, as per the manufacturer's recommendation to avoid latex gloves during mixing of the base and catalyst. The mixed material was then placed in the tray (DC Dental, Germany). Another operator loaded the tray with light body material, covering the putty and filling the teeth sulcus. The tray was then placed over the model to allow simultaneous setting of the materials. Each impression was left to set for 12 min, which was double the recommended setting time to ensure complete polymerization of the material. The impressions were then removed from the master model by first pulling the anterior part, followed by the posterior part. This process was repeated seven times for each of the master models (*n* = 7).

### 2.3 Two‐Step Method

In the two‐step technique, a 1.5 mm polyethylene spacer was positioned on top of the abutments and edentulous ridge. Initially, an impression was made using putty material on the model, along with the spacer, and left to set for 12 min. After removing the spacer, the tray was filled with light body material and a final impression was taken from the model (Shalileh et al. 2023). Another 12 min was considered for the complete polymerization of the light body material. The tray was removed with steps similar to the one‐step technique impression. This process is repeated seven times for each master model (*n* = 7).

**Figure 2:**
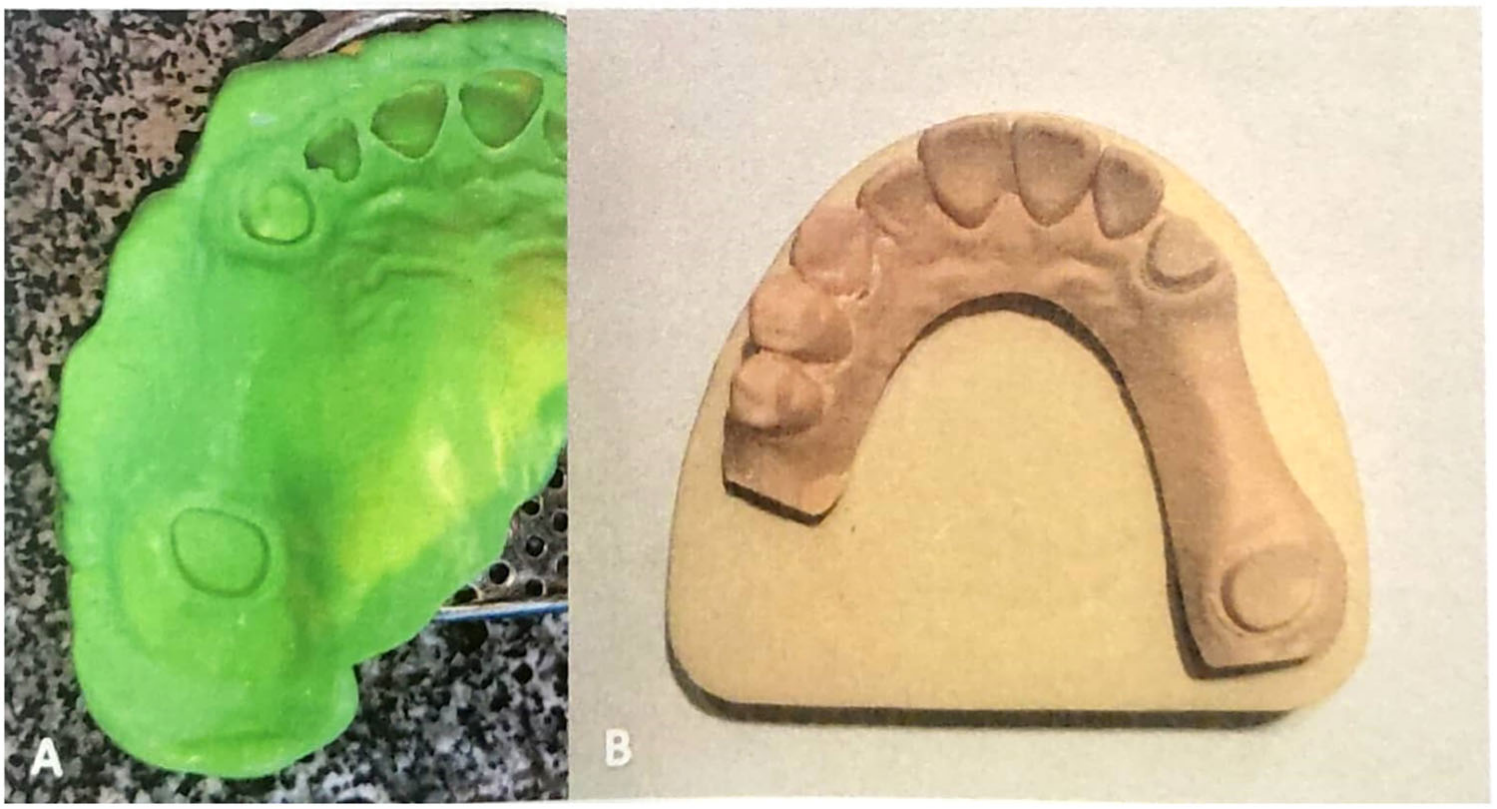
(A) Final impressions using heavy putty and light body A‐Silicone impression material taken with a perforated metal stock tray. (B) Stone cast obtained from the impression.

The impressions were captured using a spacious perforated metal stock tray (GC, USA) at a room temperature of 25°C. Afterward, they were left undisturbed for a duration of 1 h before pouring. Each of the master models was assigned Group A (one‐step technique) or Group B (two‐step technique) labels. Zhermack Elite Stone Type IV dental stone plaster was used to pour the impressions. As per manufacturer's instructions, a powder‐to‐water ratio of 20 mg per 100 mL was first mixed by a hand spatula for 10 s then mechanically mixed under vacuum for another 20 s. All the mixing process was performed on a vibrator by a single operator. The poured impressions were allowed to set for an hour, then the impressions were removed and a 48 h rest was given to the casts to reach a dimensionally stable state. A base with the same Type IV stone plaster (Neelkanth Green, Germany) was made for each dental cast (Figure 2).

**Figure 3:**
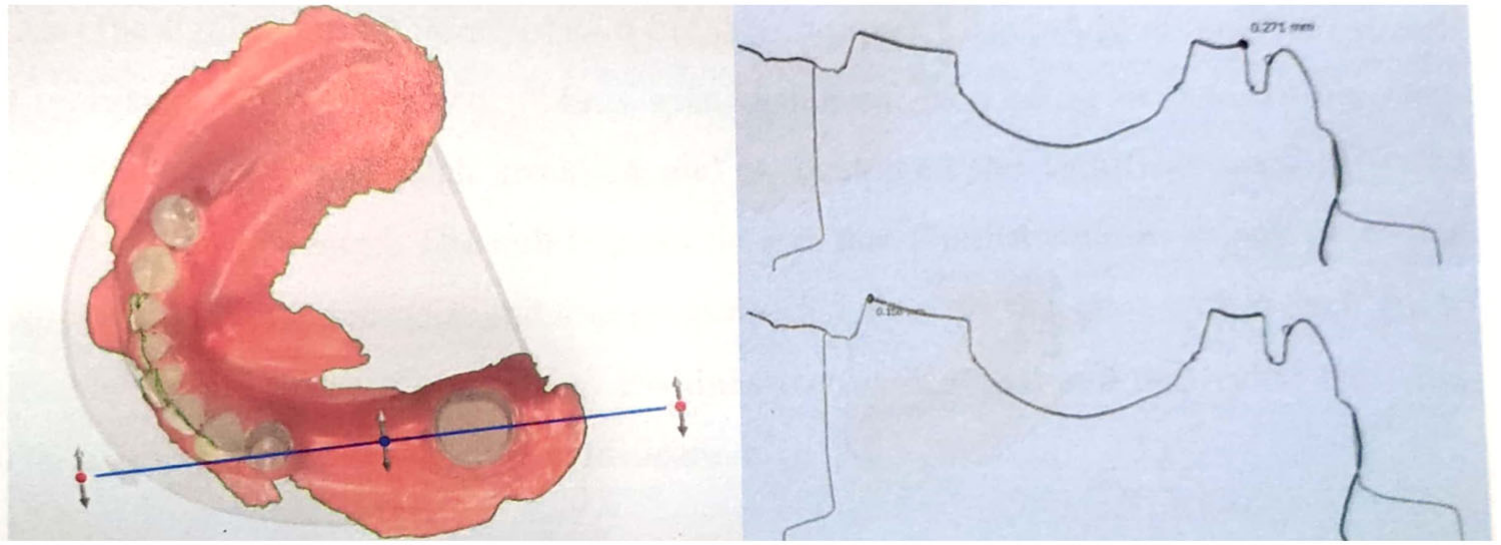
Superimposition of impression cast scan over the master model with cross‐sectional plane.

A total of 56 casts were acquired. Each cast underwent scanning using the 3Shape D810 scanner and was then overlaid onto its corresponding master model, which had been previously scanned. Subsequently, the SPL file was generated. The superimposition process was carried out utilizing the 3Shape Dental System software, with three teeth serving as landmarks: the rearmost tooth located away from the edentulous area and abutments, the foremost tooth, and the tooth positioned between the first and second landmarks. Cross‐sections were created on each abutment, in a mesiodistal direction, by referencing the markings on the master model. The maximum variations between the outline of the master model and the outline of the cast on each abutment were measured and documented in a table. Figure 3 illustrates the superimposition of one of the samples.

### 2.4 Statistical Analysis

The Excel spreadsheet contained the distance data and was then imported into a statistical program, IBM SPSS Statistics v20 (SPSS Inc, USA). The Kolmogorov–Smirnov test was employed to determine the normal distribution of the data. This test is particularly useful for assessing whether a sample comes from a specific distribution—in this case, the normal distribution. It compares the empirical distribution function of the sample with the cumulative distribution function of the normal distribution. A significant result (*p* < 0.05) indicates that the data does not follow a normal distribution, which is crucial for deciding on subsequent statistical analyses.

Following this, the Mann–Whitney *U* test was utilized to assess significant differences between the two techniques being compared. This nonparametric test is appropriate when the assumptions of normality are violated, as it does not require normally distributed data and is based on ranks rather than raw data values. The choice of this test aligns with our findings from the Kolmogorov–Smirnov test, ensuring that we utilized a method suitable for our data's characteristics.

Within each Group, A and B, Friedman's test was conducted to evaluate the significance of different interabutment distances while accounting for our sample size. This nonparametric test is particularly useful for comparing multiple related groups. It assesses whether there are any differences in treatments across multiple test attempts, making it suitable given our repeated measures design. In our study, we ensured that each group had sufficient power to detect differences if they existed. A sample size calculation was performed before data collection to establish that our sample sizes would provide adequate power (typically 80% or greater) to detect significant effects based on expected effect sizes.

## 3 Results

**Figure 4:**
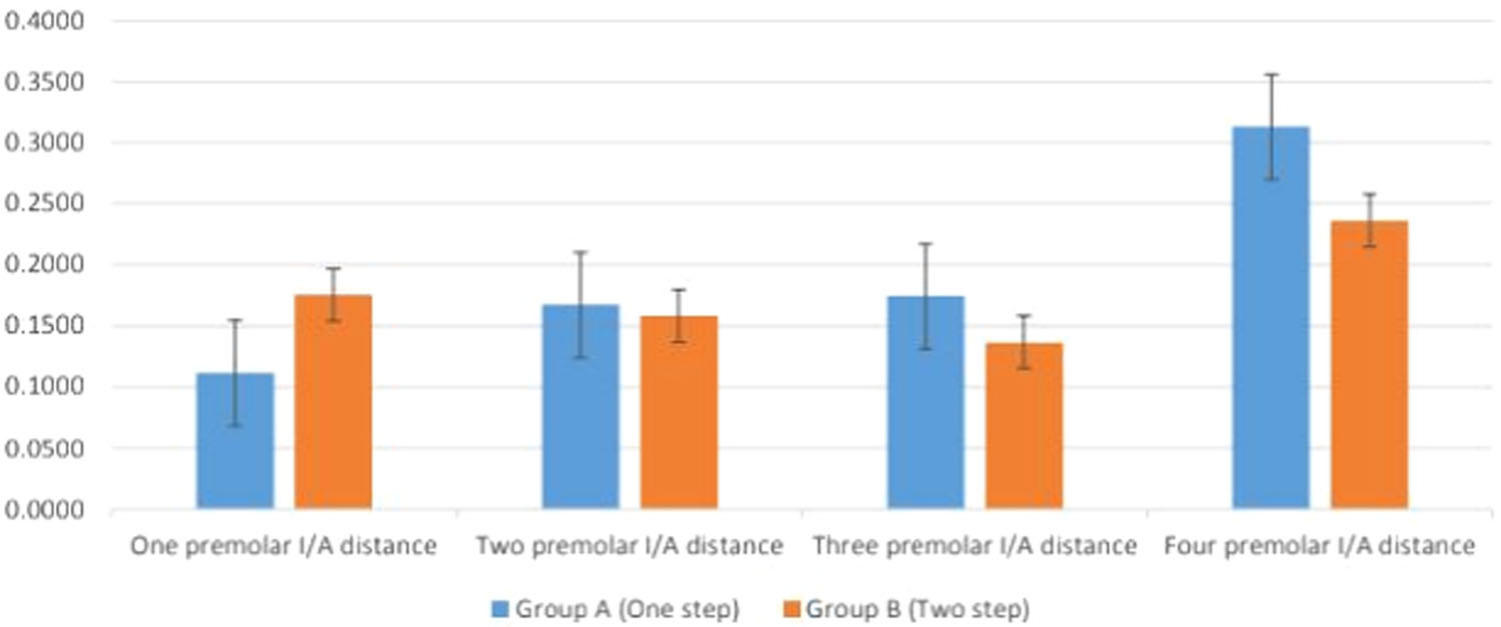
Bar chart comparing the mean differences in different interabutment (*I*/*A*) distances in one‐step and two‐step impression techniques.

Table 1 shows the mean and standard deviation (SD) values of superimposition one‐step and two‐step impression techniques on different interabutment distances. Additionally, Figure 4 represents the mean differences between Groups A and B. The higher mean difference value represents a lower accuracy of the impression method. Accordingly, the differences between Groups A and B in three premolar distances were significant (*p* < 0.05), with the two‐step impression technique being found to be more accurate.

**Table 1:** Mean and standard deviation of intera‐butment distances with one‐step and two‐step impression techniques with *p* value of each group Mann–Whitney test.

Interabutment distance	Group A (Mean ± SD of interabutment)	Group B (Mean ± SD of interabutment)	*p* value
One premolar	0.111 ± 0.064	0.175 ± 0.098	0.225
Two premolar	0.167 ± 0.070	0.158 ± 0.112	0.482
Three premolar	0.174 ± 0.032	0.136 ± 0.031	0.047
Four premolar	0.313 ± 0.127	0.236 ± 0.065	0.277
*p* value	0.01	0.18	

In Group A, which followed the one‐step impression technique, a significant difference was observed in the previous test. Subsequently, a pairwise comparison was conducted to analyze the interabutment distances between the one premolar and two premolar, three premolars, and four premolars. The results indicated that there was a significant difference in the interabutment distances between the one premolar and two premolars, three premolars, and four premolars (*p* = 0.006) (Table 2).

**Table 2:** Friedman pairwise analysis in one‐step impression technique group. Significant differences were found to be between one premolar and four premolar interabutment distances. The significance level is *p* = 0.05.

Comparison	Test statistic	Standard error	Significance	Adjusted significance
One premolar/two premolars	−0.923	0.691	0.178	1
One premolar/three premolars	−1.072	0.691	0.121	0.732
One premolar/four premolars	−2.281	0.691	0.001	0.006
Two premolars/three premolars	−0.144	0.691	0.836	1
Two premolars/four premolars	−1.353	0.691	0.049	0.295
Three premolars/four premolars	−1.211	0.691	0.078	0.471

## 4 Discussion

The physical properties of impression materials directly affect the impression. Therefore, the accuracy and stability of an impression are important factors that influence long‐term clinical success. The ability to accurately record the tooth preparation during the impression stage is crucial for the production of suitable crowns and bridges. Impression materials that exhibit good stability and dimensional accuracy are essential for capturing fine details of hard tissues and obtaining acceptable restorations (Perry 2013). Accordingly, in the present study, the accuracy of the one‐step and two‐step impression techniques was evaluated in different interabutment distances. Moreover, the null hypothesis, which suggests that the distribution among the various edentulous areas is equal, was rejected by the one‐step impression group. To put it differently, a statistically significant distinction (*p* = 0.01) was observed among the different edentulous areas when using the one‐step impression technique. Conversely, the two‐step impression technique did not yield any significant differences in the various edentulous span areas (*p* = 0.18).

The initial hypothesis stated that the accuracy of the one‐step and two‐step impression techniques is not affected by the interabutment distance. Furthermore, the second hypothesis suggested that the accuracy of the impression results is not influenced by the one‐step and two‐step techniques. However, the results obtained do not support our first null hypothesis, as it was found that the interabutment distance among the three premolar distances can indeed impact the accuracy of the impression. The findings revealed that the two‐step impression technique produced a smaller gap and greater accuracy across the three premolar distances. The average interabutment distance in this technique was measured to be 0.136 μm, whereas it was 0.174 μm in the one‐step technique. In simpler terms, the two‐step impression technique resulted in stone casts that exhibited a smaller dimensional difference when compared to the master model, thus leading to more precise final impressions. This can be attributed to the fact that the putty material acts as a custom tray for the light‐body material, which has a lower viscosity. Basapogu et al. (2016) presented contrasting findings in their investigation. They illustrated that the precision of the one‐step impression method was comparable to that of the two‐step technique. Since the impression materials utilized in both studies differed, the variance observed in the outcomes could be attributed to the materials and methodologies employed. In the two‐step technique, a polyethylene spacer was used to create space. Additionally, reference points were identified on the two abutments, and the distances between these points and between points on the same abutment were assessed. Tan et al. (2019) demonstrated that reducing the distance between dental abutments enhanced the accuracy of digital impressions, whereas this parameter did not impact the conventional impression technique. Thanasrisuebwong et al. (2021) showed that the error of impression accuracy and precision increased with the increasing interdental spaces. Basaki et al. (2017) showed that the difference in distance between the tooth models has no effect on the impression accuracy. Kim and Kim (2018) reported an inevitable change in the internal and marginal dimensions of the abutments adjacent to a pontic. This discrepancy was decreased in two‐step technique from one premolar to two premolar followed by three premolar edentulous span groups, a sudden increase was observed in the four premolar space group.

Additive PVS impression materials have two types of viscosities to match different impression needs (Menaga et al. 2024). The light body has the lowest viscosity and is placed on both hard and soft tissues, resulting in the detection of fine details of the surface of the tooth preparation. However, the light body has insufficient dimensional stability to maintain its shape during impression manufacturing (Kumari and Nandeeshwar 2015). PVS has proved to possess excellent chemical and mechanical properties for conventional impression of fixed dental prostheses among other dental impression materials (Apinsathanon et al. 2022). The one‐step and two‐step impression techniques are the most frequently utilized methods with this particular type of impression material (Jafari et al. 2024). The precision of both the one‐step and two‐step techniques has been a subject of contention in research. Several studies have indicated that the two‐step technique yields more accurate impressions. Pande and Parkhedkar (2013), on the other hand, reported that the one‐step impression technique with A‐silicone impression material exhibited superior dimensional accuracy compared to the two‐step technique. However, some other research reported no significant difference between the two aforementioned impression techniques (Afshari et al. 2020; ShivaKumar et al. 2020).

Despite observing notable disparities between the two methods, these variances may not hold clinical significance. The rise in interabutment distance can be attributed to the impression material adhering to the adhesive‐coated tray. Due to the limitations imposed by an efficient adhesive during the setting process, the abutments in the final cast may appear to be further apart than they were in the original model. Only a few studies have compared PVS to the additional silicone using various impression techniques (Dad et al. 2022; Naumovski and Kapushevska 2017; Levartovsky et al. 2014). Hence, it is advisable to conduct additional research to compare vinyl siloxane ether with various impression materials utilizing diverse impression techniques. The current investigation revealed that the two‐step impression technique exhibited a significantly higher level of accuracy than the one‐step impression technique.

The selection of impression technique is impacted by numerous factors such as ease of execution and time. Each technique possesses its own set of advantages and disadvantages (Omer et al. 2024). The one‐step impression technique offers simplicity in execution, utilizes a smaller amount of material, and requires the tray to be placed in the patient's mouth only once. On the other hand, the two‐step impression technique has been proven to be more accurate compared to the one‐step technique (Moreira et al. 2015; Levartovsky et al. 2014).

One major disadvantage of the one‐step impression technique is the potential displacement of the light‐body material by the putty material when the tray is placed over the dental abutments. However, this issue can be avoided in the two‐step impression technique by ensuring complete polymerization of the putty material. Furthermore, the one‐step dental impression technique is also associated with a higher likelihood of bubble formation, which is considered a drawback (Jamshidy et al. 2016).

The previous studies have yielded conflicting results regarding the effect of impression techniques. This conflict could be attributed to the difference in the materials, methods, and evaluation approaches. Both impression techniques have their own strengths and drawbacks. However, the accuracy of the impression techniques was also evaluated in the increasing interabutment distances for each impression technique group (Jamshidy et al. 2016; Aggarwal et al. 2019). The effect of interabutment distance was found to be statistically significant in the results of the one‐step impression technique group. However, in the two‐step technique, the effect of interabutment distance was not statistically significant.

There are certain limitations to acknowledge in the present study. The study was conducted using an in vitro design, which means that it was not possible to evaluate the impact of factors such as blood, saliva, oral temperature, and the clinical setting environment on the accuracy of impression techniques. Additionally, due to our chosen methodology, this study focused only on assessing one‐step and two‐step impression techniques based on interabutment distances. However, it is important to note that various other factors, such as fabrication patterns, flasking, and the types of materials used, can also influence the accuracy of impressions. Therefore, further clinical studies are necessary to gain a better understanding of the role of impression techniques and the various factors that can affect their accuracy.

## 5 Conclusion

In this study, we found that the two‐step impression technique yielded more precise impressions compared to the one‐step technique in cases where the edentulous interabutment distance corresponded to three premolar teeth. Although there was an overall improvement in accuracy with the two‐step technique, this difference was not statistically significant. Conversely, the accuracy of the one‐step technique was significantly influenced by the distance between the dental abutments; as the distance increased from one premolar to four premolars, the accuracy of the final impression decreased.

These findings have practical implications for dental practitioners when selecting impression techniques based on interabutment distances. For scenarios involving shorter distances (such as those corresponding to three premolars), the two‐step technique may be preferred for its enhanced precision. However, practitioners should be cautious when using the one‐step technique for greater distances, as this may lead to decreased accuracy in final impressions. Overall, understanding these dynamics can aid clinicians in making informed decisions about which impression technique to employ, ultimately improving patient outcomes.

## Author Contributions

*Data collection:* Seyyed Ahmad Ghoraishian, Mohsen Khataminia, and Zahra Shahramian. *Data analysis:* Maryam Zare, Mina Mohaghegh, and Mohsen Khataminia. *Manuscript revision:* Maryam Zare, Mina Mohaghegh, and Seyyed Ahmad Ghoraishian. *Manuscript writing:* Zahra Shahramian and Mohsen Khataminia. *Statistical analysis:* Mohsen Khataminia.

## Ethics Statement

This study has received approval from the ethical committee of Shiraz University of Medical Sciences (IR.SUMS.DENTAL.REC.22471).

## Conflicts of Interest

The authors declare no conflicts of interest.

## Data Availability

The data that support the findings of this study are available on request from the corresponding author. The data are not publicly available due to privacy or ethical restrictions.
